# Localized Dielectric Loss Heating in Dielectrophoresis Devices

**DOI:** 10.1038/s41598-019-55031-y

**Published:** 2019-12-12

**Authors:** Tae Joon Kwak, Imtiaz Hossen, Rashid Bashir, Woo-Jin Chang, Chung Hoon Lee

**Affiliations:** 1 Department of Mechanical Engineering, University of Wisconsin-Milwaukee, Milwaukee, Wisconsin 53211 USA; 2 Department of Electrical and Computer Engineering, Marquette University, Milwaukee, Wisconsin 53233 USA; 3 Department of Bioengineering, Micro and Nanotechnology Laboratory, University of Illinois at Urbana Champaign, Champaign, Illinois 61801 USA; 4 School of Freshwater Sciences, University of Wisconsin-Milwaukee, Milwaukee, Wisconsin 53204 USA; 5 Nanoscale Devices Laboratory, Marquette University, Milwaukee, Wisconsin 53233 USA

**Keywords:** Sensors and probes, Electronics, photonics and device physics

## Abstract

Temperature increases during dielectrophoresis (DEP) can affect the response of biological entities, and ignoring the effect can result in misleading analysis. The heating mechanism of a DEP device is typically considered to be the result of Joule heating and is overlooked without an appropriate analysis. Our experiment and analysis indicate that the heating mechanism is due to the dielectric loss (Debye relaxation). A temperature increase between interdigitated electrodes (IDEs) has been measured with an integrated micro temperature sensor between IDEs to be as high as 70 °C at 1.5 MHz with a 30 *V*_*pp*_ applied voltage to our ultra-low thermal mass DEP device. Analytical and numerical analysis of the power dissipation due to the dielectric loss are in good agreement with the experiment data.

## Introduction

Temperature is the basic physical property in living cells that controls biological responses and cellular functions^1,2^. Thus, the amount of energy expenditure required for the normal functioning of the cells and organs is reflected in their temperature^3^. Moreover, cellular reactions are controlled by small temperature changes (e.g. 5.5–6 °C), and continuous heat exposure causes cell death^4,5^. Therefore, better awareness and attention to temperature are demanded in biomedical studies to diagnose or to understand the metabolism of living cells.

Dielectrophoresis (DEP) has been used in various biomedical applications such as drug delivery and discovery^6–8^, particle separation and sorting^9–11^, cell patterning^12–14^, and intermolecular force spectroscopy^15,16^ due to the benefits of strong controllability, easy operation, and high efficiency. Also, the contact-free feature of DEP results in less damage to the particles, compared with conventional microfluidic and mechanical techniques^17^. The spatial gradient of the non-uniform electric field enables the manipulation of dielectric particles by DEP. Typically, the DEP is generated by two or more electrodes integrated on a substrate or an insulating structure. However, manipulating sub-micron particles and low dielectric property bioparticles requires relatively high applied potentials to generate an electric field sufficient to manipulate particles, and the high potentials may cause heating, which may result in a temperature rise inside the device. There are two heating mechanisms during DEP device operation. One is the Joule heating caused by the conductivity of the media. The other is the dielectric loss heating of the dielectric material between the DEP electrodes. While Joule heating is dominant when electrical current is flowing between bare electrodes exposed to the conductive media, the dielectric loss heating is dominant when the electrodes are electrically insulated from the media and the conductivity of the media is relatively low.

Joule heating is a function of electrical current and the electrical conductivity of the media and is independent of the operation frequency of a DEP device. On the other hand, the dielectric loss heating is a function of the operation frequency. At operation frequencies less than 500 kHz, the dielectric loss heating is negligible in our device. At frequencies higher than 500 kHz, the dielectric loss heating gradually increases and saturates at about 10 MHz. At the saturation frequency, the maximum dielectric loss can be approximated with the Debye relaxation time constant, *τ*_*d*_. *τ*_*d*_ is a function of the dielectric constant of the material of the substrate or media. In a typical DEP operation frequency range, the Debye relaxation time constant of the dielectric constituent of the device is dominant over that of the media because the media is most likely water (buffer), which has a much larger Debye relaxation time constant than typical substrates (glass, polymer, and silicon-based materials). The dielectric loss heating presented in this paper at a fixed operating frequency can explain the increased temperature in microchips with a high spatial resolution^18^, the reduction of the risk of material breakdown in the Joule heating method^19^, and the heating of a droplet in a microchip to perform DNA melting and detection of single-base mismatches^20^.

Numerical and experimental analyses of Joule heating in DEP devices have shown the temperature effects on the conductivity of the medium, electrothermal flow, and DEP trapping forces^21–23^. Several DEP studies tried to minimize the Joule heating by using low-conductivity buffer solutions^9,24^, increased or decreased device geometric scale^25–27^, low voltage operation with three-dimensional structure^28^, and coating electrodes with insulation layer^29–31^. In addition, various methods have been applied to measure the heat generation within the DEP device. Commercially available thermometers^16,32,33^ were used to measure spatially averaged temperatures in DEP devices by attaching them to the chip surface. These average temperature measurements are suitable for a large area, rather than a micro-scale local area. A thermo-dependent fluorescent dye^24,34,35^ was used to measure temporal and spatial temperature measurements with less than 1 °C resolution in dielectrophoretic devices; however, dye-based measurement methods could leave chemical residues in the fluid channel. This might affect the metabolism of biological cells due to environmental sensitivity, and it was difficult to measure the temperature of these particles in real time. Impedance and resistance measurements^24,36^ were used to measure temperature changes and dielectric losses in the electric field as a function of frequency in real time; however, since the measurement electrodes were in contact with media, only the Joule heating induced from the conductivities of the media was focused on.

A resistive temperature detector (RTD) is a temperature sensor widely used to measure temperature changes in microchip devices, owing to its integrability, high accuracy, high linearity, and fast response time^37–39^. Integrated RTDs have been used to monitor local temperatures in DEP devices. For example, Schwamb *et al*.^40^ used a platinum RTD to measure the thermal conductivity of reduced graphene oxide flakes deposited on a DEP device. Bhattacharya *et al*.^41^ monitored the polymerase chain reaction thermal cycling efficiency and heating and cooling rates of captured bacteria on a DEP device. Gallo-Villanueva *et al*.^42^ used a copper RTD to measure Joule heating effects in insulator-based DEP devices.

While the temperature increase during DEP is well known, the previous studies with a DEP device have focused on mainly temperature measurements and considered that the temperature increase was due to Joule heating without detailed justification. However, the dielectric loss from the dielectric material between electrically insulated IDEs is found to be the main source of the temperature increase in DEP devices.

In this paper, considerable heat generation due to the dielectric loss in a DEP device is presented. While Joule heating is independent of the operating frequency, the dielectric loss heating is a function of the operating frequency. The dielectric loss is due to the imaginary part, *ε*″, of the dielectric constant of materials. *ε*″ is a function of frequency, and the power loss due to the dielectric loss is also a function of frequency. The power loss is dissipated as heat in the device. The Debye dielectric loss theory agrees very well, *R*^2^ = 0.9998, with the measured temperature increase on the device.

Our device for this study consists of interdigitated electrodes (IDEs) commonly used in DEP. A thin nickel film RTD is integrated between the IDEs. The temperature increase due to the dielectric loss can be as high as 70 °C without any liquid in the DEP device when 30 *V*_*pp*_ is applied to the IDEs. With a buffer solution and a 30 *V*_*pp*_ voltage applied to the IDEs, the temperature increase is ~10 °C, which is high enough to affect biological states of biological entities.

## Materials and Methods

**Figure 1:**
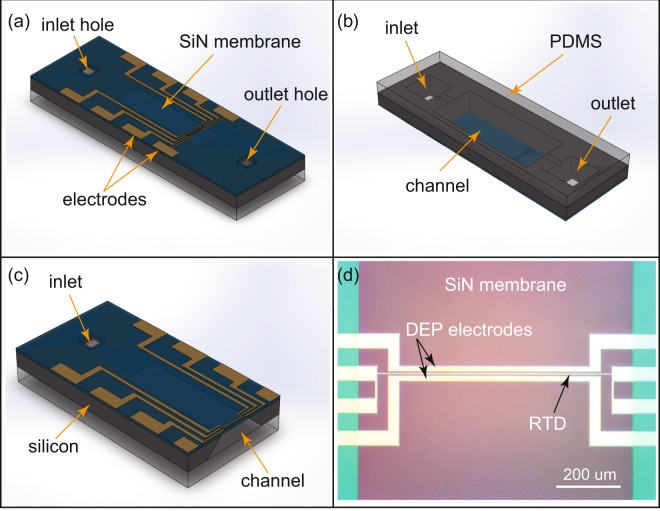
Device overview. (**a**) Isometric view of the device. IDEs, RTD, and electrical contacts are integrated on the top surface of the SiN membrane. Inlet/outlet tubing is mounted on the inlet/outlet holes (not shown). IDEs, RTD, and electrical contacts are electrically isolated from the channel by the 500 nm thick SiN membrane. (**b**) Channel side of the device. Silicon is etched to form the main channel and inlet/outlet with feed through. A 1 mm thick PDMS is placed to complete the channel. (**c**) Cross-sectional view of the device showing the channel and IDEs/RTD. (**d**) Close-up optical photograph of the top view of IDEs and RTD on the SiN membrane.

Figure 1 shows the DEP device with an integrated nickel thin film RTD. The IDEs and RTD are integrated on a 500 nm thick SiN membrane. The electrodes and the RTD are made of a 25 nm thick nickel film. The device is fabricated with a standard micromachining technique, and the detailed fabrication method is described elsewhere^43–46^. In brief, the inlet, outlet, and channel are formed by 30% w/w KOH etch at 60 °C bath temperature. The DEP electrode and the RTD are integrated on the SiN membrane side with a 25 nm thick metal film evaporated by a thermal evaporator and patterned by Ni etchant (Transene Company Inc.). The microfluidic channel is formed by placing a thin (~1 mm thick) PDMS film on the channel side of the Si chip.

The two IDEs of the DEP device are connected to a voltage amplifier (TREK 2100HF), which amplifies a function generator (Keysight 33220 A) signal. The output voltage of the amplifier is monitored by an oscilloscope (Keysight DSOX2004A). The RTD is configured as a 4-wire measurement with a Keithley 2400 source/meter unit. The RTD resistance at room temperature (18 °C) is 3104 Ω. The temperature coefficient of resistance (TCR) is 0.0026/°C^37^. The RTD is biased with 0.1 mA DC current. The RTD has 0.002 K temperature resolution^46^. All the electronics and data acquisition are controlled and collected by a LabView program.

**Figure 2:**
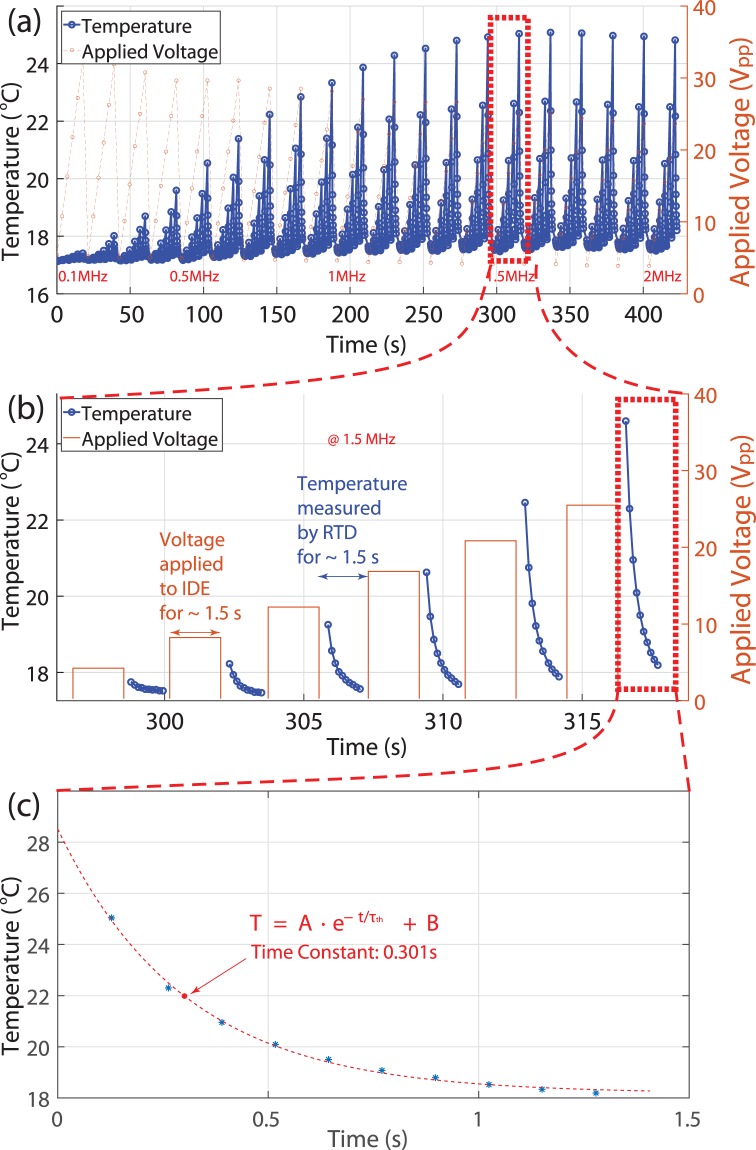
Measured temperature data as a function of time. The channel is filled with PBS. (**a**) Measured temperature data. (**b**) Close-up view of the dotted box in (**a**). Voltages are applied to IDEs for 1.5 s, and then the RTD measures the temperature for 1.5 s. The measurement cycle is repeated for the frequency range from 100 kHz to 2 MHz at frequency increments of 100 kHz. (**c**) Close-up view of the dotted box in (**b**). The temperature data is fitted with $T=A\cdot {e}^{-t/{\tau }_{th}}+B$, which is the temperature cooling of a first order system. The thermal time constant, *τ*_*th*_, is 0.301 s when the channel is filled with PBS. The *τ*_*th*_ is ~0.2 s when the channel is empty.

The DEP excitation and temperature measurement are set as follows. A desired voltage of 5 *V*_*pp*_ from the amplifier at a fixed frequency of 100 kHz is applied to the DEP device for 1.5 second. Then the voltage is turned off. After this, the RTD measures the temperature for 1.5 second. The sequential DEP excitation and temperature measurement precludes any electrical interference between them. The DEP excitation and temperature measurement cycle is repeated with an increased amplifier voltage at the same frequency. For example, 10 *V*_*pp*_ at 100 kHz is applied for 1.5 seconds followed by temperature measurement for 1.5 seconds. The excitation and measurement cycle are repeated for 5 different voltages at the frequency. Once the temperature measurement for the voltage range from 5 *V*_*pp*_ to 30 *V*_*pp*_ is done, the applied frequency is increased to 200 kHz, and the temperature measurement is repeated with the same voltage range. The excitation and measurement cycle are repeated for the frequency range from 100 kHz to 2 MHz at increments of 100 kHz. The raw temperature measurement data of the DEP device filled with PBS in the channel is shown in Fig. 2(a). Figure 2(b) is a close-up of the red dot boxed area in Fig. 2(a). The temperature is measured ~0.1 second after the voltage from the amplifier is turned off. The thermal time constant, *τ*_*th*_ is shown in Fig. 2(c), which is a close-up of the red dot boxed area in Fig. 2(b). The dotted curve in Fig. 2(c) is a fit of the data with $T=A\cdot {e}^{-t/{\tau }_{th}}+B$, which can be used for a first order thermal system.

## Results and Discussions

**Figure 3:**
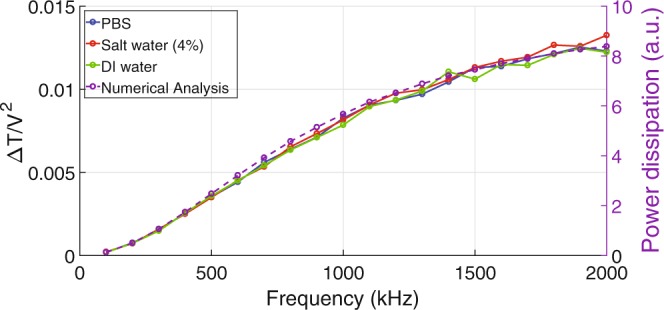
Measured Δ*T* per unit power (~*V*^2^) of PBS, Salt (NaCl) water (4%), and DI water. No obvious difference between three samples is noticeable, indicating that no Joule heating mechanism is involved. COMSOL is used to calculate the power dissipation (loss power) in the SiN dielectric located between the DEP electrodes. Since the power dissipation is proportional to the temperature, the calculated results are in good agreement with the measured data.

As shown in Fig. 2(a), the applied voltages at each frequency de due to the loading effect of the capacitive load (DEP). Therefore, the data is re-plotted as the maximum temperature increase divided by the applied voltage squared (~Δ*T* per input power) as a function of the excitation frequency as shown in Fig. 3. The Δ*T* is defined as *T*_*measured*_ − *T*_*abmient*_(18 °C). The Δ*T* per input power of three samples (DI water, salt (NaCl) water (4%), and PBS) is shown in Fig. 3. The temperature increases per unit power for all three samples are independent of the ion concentration in the media. This is another strong indication that the heating mechanism is due to the dielectric loss.

The heat generation in a dielectric material between electrodes can be analyzed with the Debye relaxation. Our DEP device is integrated on a SiN membrane. The SiN film is a good dielectric material (low electrical conductivity). The DEP device configuration can be modeled as a non-conventional capacitor consisting of exposed side by side plates with multiple dielectric materials (electrode-membrane-solution-membrane-electrode) in between.

The apparent power flow into the capacitor is,

1 $${P}_{t}=VI=j{V}^{2}\omega {C}^{\ast }=j{V}^{2}\frac{\omega A}{d}(\varepsilon ^{\prime} -j\varepsilon ^{\prime\prime} )$$

where *P*_*t*_ is the total power delivered to the capacitor, *V* is the voltage applied, C* is the complex conjugate of capacitance, *ω* is the applied angular frequency, *A* is the area of the capacitor, *d* is the distance between electrodes, *ε*′ is the AC capacity (real part of the permittivity), and *ε*″ is the dielectric loss factor (imaginary part of the permittivity).

The heat generated by the Debye relaxation is described by the dielectric loss factor,

2 $${P}_{d}={V}^{2}\frac{\omega A}{d}\varepsilon ^{\prime\prime} $$

where *P*_*d*_ is the power dissipated in the capacitor. The dielectric loss^47^ can be expressed as

3 $$\varepsilon ^{\prime\prime} =({\varepsilon }_{0}-{\varepsilon }_{\infty })\frac{\omega {\tau }_{d}}{1+{(\omega {\tau }_{d})}^{2}}$$

where, *ε*_0_ is the permittivity at frequencies below dipole relaxation, ${\varepsilon }_{\infty }$ is the permittivity at frequencies above dipole relaxation, and *τ*_*d*_ is the Debye relaxation time constant.

The power, *P*_*d*_, is dissipated in the capacitor. Since the power is applied for a given time to the capacitor, the step response of a first-order system can be used to obtain the temperature response using Fourier’s Law of Heat conduction.

The temperature increase or decrease in the capacitor by a step power input is,

4 $$\Delta T(t)={P}_{d}{R}_{t}\cdot [1-\exp (-t/{\tau }_{th})]$$

or

5 $$\Delta T(t)={P}_{d}{R}_{t}\cdot \exp \,(-t/{\tau }_{th})$$

where Δ*T*(*t*) = *T*_*cap*_(*t*) − *T*_*amb*_, *T*_*cap*_(*t*) is the temperature of the capacitor, *T*_*amb*_ is the ambient temperature, *R*_*t*_ is the thermal resistance between the capacitor and ambient, and *τ*_*th*_ is the thermal time constant. The time constant, *τ*_*th*_, can be measured directly as shown in Fig. 2(c) and has values of ~0.2 and ~0.3 second with air and PBS, respectively. The time period of the applied power to the capacitor is ~1 second. Therefore, the temperature of the capacitor reaches the steady state temperature, Δ*T*_*ss*_. The thermal time constant is obtained by fitting Eq. 5 with the data.

From the dielectric loss and the first order thermal analysis, the steady state temperature increase from the ambient temperature of the capacitor can be expressed as,

6 $$\Delta {T}_{ss}={P}_{d}{R}_{t}$$

Assuming all the dielectric loss is converted to heat, the steady state temperature can be written as,

7 $$\Delta {T}_{ss}={V}^{2}\frac{{R}_{t}A({\varepsilon }_{0}-{\varepsilon }_{\infty })}{d\,{\tau }_{d}}\frac{{({\tau }_{d}\omega )}^{2}}{1+{({\tau }_{d}\omega )}^{2}}$$

**Figure 4:**
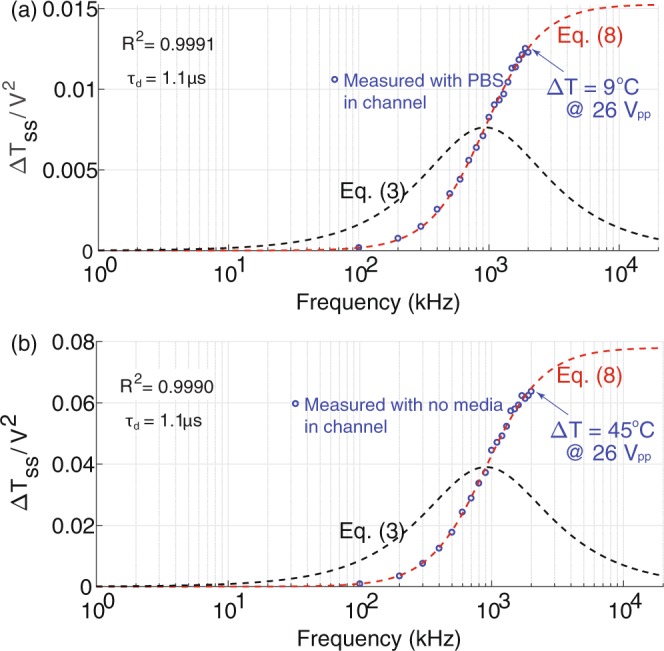
Temperature increase per applied voltage squared (unit power) due to the dielectric loss in a DEP device as a function of frequency. The maximum Δ*T* for PBS and no media in the channel is 9 °C and 45 °C, respectively, at an applied voltage of 26 *V*_*pp*_. The maximum Δ*T* with PBS in the channel is lower due to the larger thermal mass of PBS compared to that of air. (**a**) The temperature is measured with PBS solution in the channel. The measured data is in good agreement with the dielectric loss analysis (Eq. 8). The blue circles are measured data. The red dotted line is the power dissipated in the capacitor (Eq. 8). The black dotted line is the dielectric loss factor (Eq. 3). The regression of the data fit is higher than 0.999. (**b**) The same plot with no media in the channel (empty). The data clearly shows that the temperature increase is due to the SiN dielectric loss since no media is in the channel.

Δ*T*_*ss*_/*V*^2^ as a function of frequency (*f* = *ω*/2*π*) is shown in Fig. 4.

The data is fit with the dielectric power loss equation,

8 $$y=a\frac{{(b\cdot f)}^{2}}{[1+{(b\cdot f)}^{2}]}$$

with *a* = 0.0152 and *b* = 1.1 · 10^−6^. *a* is ${R}_{t}A({\varepsilon }_{0}-{\varepsilon }_{\infty })/d{\tau }_{d}$ in Eq. 7. *b* is the Debye time constant, *τ*_*d*_, in seconds. The regression, *R*^2^, is higher than 0.999, indicating the analysis is in good agreement with the data.

To evaluate the dependencies of the heat generated by the dielectric loss and the power dissipation on applied potentials and frequencies, we analyzed the thermal characteristics of the DEP device with finite element analysis (FEA) numerical simulations using COMSOL Multiphysics software (v5.2, COMSOL AB, Stockholm, Sweden) with the AC/DC and the heat transfer modules.

The materials used in the simulation were a Ni film for the electrodes, a SiN membrane for the insulation layer, and water as media (the same as used in the experiments). The basic properties from the COMSOL Multiphysics Materials Library were used. AC electrical potentials of 100 kHz to 2 MHz were applied to the electrodes. While a constant value of the real part of the relative permittivity (*ε*′ from Eq. 1) at different frequencies was assigned, the values of the imaginary part of the relative permittivity (*ε*″ from Eq. 3) at different frequencies were assigned to the SiN layer. The geometry used in the simulation was identical to the experimental setup. The simulation covered around 100 *μ*m × 500 *μ*m area on the insulation layer for efficient computation. The multi-physics equations were solved by the frequency-transient analysis for 4.45 × 10^6^ degrees of freedom with a minimum element size of 2 *μ*m.

**Figure 5:**
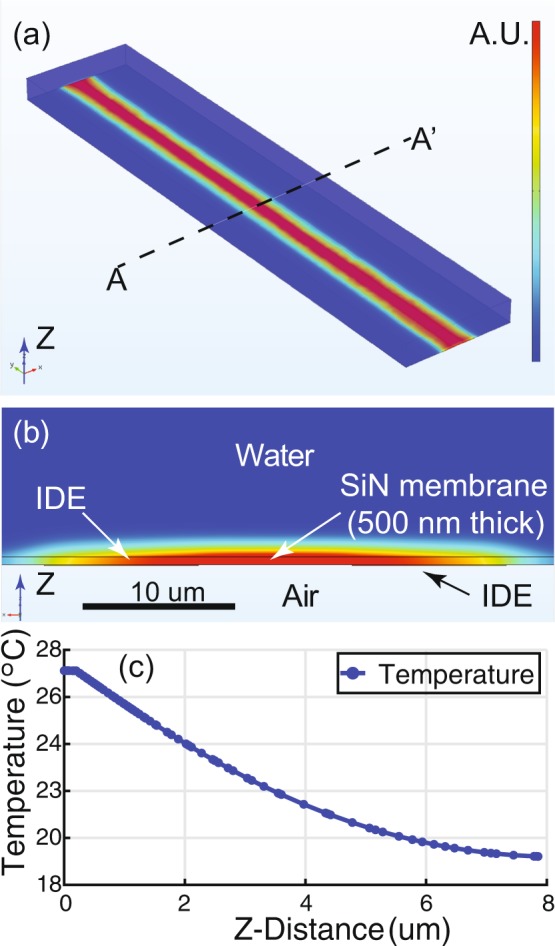
COMSOL simulation results. As shown in Fig. 3, the numerical analysis agrees with the measured results. (**a**) A perspective view of the DEP device in Fig. 1(d). The temperature increase between the IDEs is shown. (**b**) Cross-sectional view along AA’ in (**a**). The highest temperature increase occurs on the SiN membrane between the IDEs. (**c**) Temperature profile from the center of the SiN membrane (0 *μ*m) to water in the z-direction.

**Figure 6:**
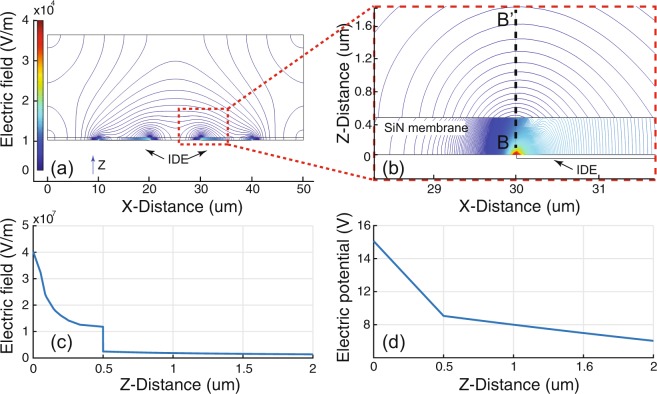
Numerical simulation of the electric field and potential in the DEP device. (**a**) Electric field distribution. (**b**) Enlarged electric field distribution of the red box area in (**a**). (**c**) Electric field along BB’ in (**b**). (**d**) Electric potential along BB’.

The three-dimensional simulations successfully demonstrate the heat generation in the device when an AC signal is applied to the IDEs. The results are shown in Fig. 5. Figure 5(a) shows a perspective view of the temperature increase in the device. We assume that the power dissipation due to the dielectric loss in Fig. 4 is mostly converted to heat between the IDEs in Fig. 5, as was previously approximated by the first order thermal system using Fourier’s Law of Heat Conduction in the analytical analysis. This result is also clearly shown in Fig. 5(b), which is the cross-sectional view of the middle of the device in Fig. 5(a). The simulation results show clearly that the highest temperature increase occurs on the SiN membrane between the IDEs. Figure 5(c) is the numerically calculated temperature profile from the center of the SiN membrane surface to the media in the z-direction. The temperature in the microchannel gradually decreases along the z-direction from the surface of the SiN membrane, which is heated by the dielectric loss. The temperature difference between the SiN membrane and the top of the microchannel (~20 *μ*m away from the membrane) is about 9 °C. As shown in Fig. 6, the strongest electric field (most potential drop) is within the SiN membrane near the electrodes. The potential drop in the media is much less than in the SiN membrane. Therefore, the Joule heating in the media is a secondary effect in our device configuration.

## Conclusions

The resistive temperature detector based temperature measurement method and the Debye relaxation analysis find that significant heat is generated when an alternating voltage is applied to the electrically insulated interdigitated electrodes based dielectrophoretic device. The experimental results are in good agreement with the theoretical and numerical calculations.

The experimental results and analysis indicate that the origin of the heat generation in the DEP devices with electrically insulated electrodes is the Debye relaxation rather than Joule heating.

The approaches to avoid heating in the past studies using DEP have mainly tried to reduce Joule heating; however, we suggest that the dielectric characteristics of material have to be considered in order to reduce the dielectric loss and to reduce heat generation in microfluidic devices.

The heat generation from the Debye relaxation is highly localized between the IDEs as shown in Fig. 5 and is more reliable than that from Joule heating, which causes the electromigration in a heating element for micro/nano-scale devices.

The localized dielectric heating process can be used as a powerful tool in a wide range of biological studies, because the heating can be easily controlled by the design, driving voltage/frequency, and materials used in the micro/nano-scale devices.
